# Optimizing adipogenic transdifferentiation of bovine mesenchymal stem cells: a prominent role of ascorbic acid in *FABP4* induction

**DOI:** 10.1080/21623945.2020.1720480

**Published:** 2020-01-29

**Authors:** Sandra Jurek, Mansur A. Sandhu, Susanne Trappe, M. Carmen Bermúdez-Peña, Martin Kolisek, Gerhard Sponder, Jörg R. Aschenbach

**Affiliations:** a Institute of Veterinary-Physiology, Freie Universität Berlin, Berlin, Germany; b Department of Veterinary Biomedical Sciences, PMAS-Arid Agriculture University, Rawalpindi, Pakistan; c Nursing Faculty, Autonomous University of Queretaro, Querétaro City, Mexico; d Division of Neurosciences, Biomedical Center Martin, Jessenius Faculty of Medicine in Martin, Comenius University in Bratislava, Martin, Slovakia

**Keywords:** Adipocytes, adipose tissue, fatty acid binding protein, lipid droplets, animal models

## Abstract

Adipocyte differentiation of bovine adipose-derived stem cells (ASC) was induced by foetal bovine serum (FBS), biotin, pantothenic acid, insulin, rosiglitazone, dexamethasone and 3-isobutyl-1-methylxanthine, followed by incubation in different media to test the influence of ascorbic acid (AsA), bovine serum lipids (BSL), FBS, glucose and acetic acid on transdifferentiation into functional adipocytes. Moreover, different culture plate coatings (collagen-A, gelatin-A or poly-L-lysine) were tested. The differentiated ASC were subjected to Nile red staining, DAPI staining, immunocytochemistry and quantitative reverse transcription PCR (for *NT5E, THY1, ENG, PDGFRα, FABP4, PPARγ, LPL, FAS, GLUT4*). Nile red quantification showed a significant increase in the development of lipid droplets in treatments with AsA and BSL without FBS. The presence of BSL induced a prominent increase in *FABP4* mRNA abundance and in FABP4 immunofluorescence signals in coincubation with AsA. The abundance of *NT5E, ENG* and *THY1* mRNA decreased or tended to decrease in the absence of FBS, and ENG was additionally suppressed by AsA. DAPI fluorescence was higher in cells cultured in poly-L-lysine or gelatin-A coated wells. In additional experiments, the multi-lineage differentiation potential to osteoblasts was verified in medium containing ß-glycerophosphate, dexamethasone and 1,25-dihydroxyvitamin D_3_ using alizarin red staining. In conclusion, bovine ASC are capable of multi-lineage differentiation. Poly-L-lysine or gelatin-A coating, the absence of FBS, and the presence of BSL and AsA favour optimal transdifferentiation into adipocytes. AsA supports transdifferentiation via a unique role in *FABP4* induction, but this is not linearly related to the primarily BSL-driven lipid accumulation.

**Abbreviations**: AcA: acetic acid; AsA: ascorbic acid; ASC: adipose-derived stem cells; BSL: bovine serum lipids; DAPI: 4´,6-diamidino-2-phenylindole; DLK: delta like non-canonical notch ligand; DMEM: Dulbecco’s modified Eagle’s medium; DPBS: Dulbecco’s phosphate-buffered saline; ENG: endoglin; FABP: fatty acid binding protein; FAS: fatty acid synthase; GLUT4: glucose transporter type 4; IBMX: 3-isobutyl-1-methylxanthine; LPL: lipoprotein lipase; MSC: mesenchymal stem cells; α-MEM: α minimum essential medium; NT5E: ecto-5ʹ-nucleotidase; PDGFRα: platelet derived growth factor receptor α; PPAR*γ*: peroxisome proliferator activated receptor γ; RPS19: ribosomal protein S19; SEM: standard error of the mean; THY1: Thy-1 cell surface antigen; TRT: treatment; TRT-Con: treatment negative control; YWHAZ: tyrosine 3-monooxygenase/tryptophan 5-monooxygenase activation protein zeta

## Introduction

Adult stem cells are undifferentiated cells that can continue their self-renewal for extended periods of time. Upon specific stimuli, they can undergo self-transformation into specialized cell types [1]. Adipose-derived stem cells (ASC) are present in many fat depots of the body and appear as fibroblast-like cells upon culture. The International Fat Applied Technology Society has stated that the term ASC should be used for committed adipogenic progenitors (pre-adipocytes). Many tissues of human and animal origin have been employed as sources of ASC, e.g. subcutaneous [2], intraperitoneal [3], muscle [4], visceral [5] and bone marrow tissues [6]. Thus, a wide array of adult adipose tissues can serve as a pool for adipocyte progenitors. Pre-adipocytes are capable of multi-lineage differentiation into myocytes, osteoblasts, chondrocytes and adipocytes [7]. This multipotency of ASC has made them focus of much research [8]. The transformation of ASC into fully functional adipocytes is dependent upon the expression of special cellular markers. Cell surface markers such as ecto-5ʹ-nucleotidase (NT5E, CD73), Thy-1 cell surface antigen (THY1, CD90), platelet derived growth factor receptor *α* (PDGFR*α*) and endoglin (ENG, CD105) allow the discrimination of ASC from other differentiated cell types [9,10]. The major characteristics of ASC are their adherence to plastic, their production of specific cell surface markers, and their differentiation capability as described by the International Society of Cell Therapy [11]. ENG is an adhesion molecule that plays an important role in transforming growth factor β signalling, which may trigger pre-adipocyte differentiation into chondrocytes [9]. NT5E is an ecto-5ʹ-nucleotidase surface enzyme that has a role in cell-to-cell interactions [10] and that is also involved in mesenchymal stem cell immunity [12]. THY1 is a less well deﬁned ASC marker and is involved in cell-to-cell interactions [13]. PDGFR*α*, a cell surface tyrosine kinase receptor, is expressed on cells that fulfill the definition of MSC and induces many effects, like cell proliferation or transformation [14].

To date, diverse types of hormonal and chemical cocktails have been suggested for the induction and further transformation of ASC into mature adipocytes with variable success, most of them containing foetal bovine serum (FBS), biotin, pantothenic acid, insulin, rosiglitazone, dexamethasone and 3-isobutyl-1-methylxanthine (IBMX) during the induction phase [15,16]. For bovine ASC differentiation, the addition of insulin and ascorbic acid (AsA) has been used at various stages of adipocyte culture [17]. Similarly, bovine serum lipids (BSL), a triglyceride-rich cell culture supplement from bovine blood, has shown a potent effect on the differentiation of intramuscular and subcutaneous pre-adipocytes into adipocytes [18]. In the experiments described herein, we intended to specifically titrate the supplement concentrations of induction and differentiation media to the needs of bovine subcutaneous ASC. Such a model system would be very helpful for studying the regulation of lipid metabolism in dairy cows. The latter may be different to humans and monogastric animals; because dairy cows have lower blood glucose levels and drive adipogenesis in adipose tissue primarily from blood acetic acid (AcA) [19]. Therefore, the bovine ASC might be a valuable model system for comparative basic research. As we have previously shown that the adipogenesis of bovine ASC is promoted by the presence of BSL and the absence of FBS [20], we wondered whether AsA may also affect adipogenic differentiation at various levels of BSL and FBS. Furthermore, we hypothesized that, for bovine ASC, AcA is an important nutrient promoting adipogenesis and that AcA effects are dependent upon glucose availability. In addition to testing these hypotheses, the methodological aims of our study were to optimize the induction and differentiation media for bovine ASC, to determine the effects of various plastic coating materials on cellular adhesion and to verify the multi-lineage potential of the used ASC by demonstrating their transdifferentiation potential towards osteoblasts.

## Materials and methods

### Materials

FBS, BSL (Ex-Cyte), trypsin-EDTA, penicillin-streptomycin, AcA, Dulbecco’s phosphate-buffered saline (DPBS), collagen-A, and the cell culture media DMEM and complete α minimum essential medium (α-MEM) were obtained from Merck Millipore (Darmstadt, Germany). N-2-Hydroxyethylpiperazine-N’-2-ethanesulfonic acid (HEPES), amphotericin B, AsA, biotin, pantothenic acid, bovine insulin, rosiglitazone, dexamethasone, IBMX, Nile red, Triton^TM^ X-100, gelatin-A, poly-L-lysine, ß-glycerophosphate, 1,25-dihydroxyvitamin D_3_, alizarin red (sodium sulphonate salt) and D-glucose were purchased from Sigma Aldrich (Taufkirchen, Germany). Mount Fluor was supplied by BioCyc GmbH & Co. (Luckenwalde, Germany) and the fixative solution Roti-Histofix 4% was from Carl Roth (Karlsruhe, Germany). The antibodies against NT5E, THY1, delta like non-canonical notch ligand 1 (DLK1), fatty acid binding protein 4 (FABP4), glucose transporter type 4 (GLUT4) and fatty acid synthase (FAS) were from AbCam (Cambridge, UK), whereas the antibody against platelet derived growth factor receptor α (PDGFRα) was obtained from Biorbyt Ltd. (Cambridge, UK). The antibody against ENG was purchased from Thermo Scientific (Massachusetts, USA) and 4´,6-diamidino-2-phenylindole (DAPI) was obtained from Roche (Grenzach-Wyhlen, Germany). Other consumables are detailed at other places. Cell culture plates and other plastic ware were from Techno Plastic Products, Trasadingen, Switzerland or resources stated elsewhere.

### Adipose tissue assortment

Tissue harvest was in accordance with the German legislation on animal welfare; however, no Animal Use and Care approval was required because no animals were specifically reared or killed for the present experiments. Samples of bovine subcutaneous adipose tissue were aseptically collected from the neck region (2^nd^ to 3^rd^ vertebrae) of exsanguinated Holstein calves (< 11 months old) from a local abattoir. After collection, adipose tissue was washed with sterile calcium- and magnesium-containing DPBS and transferred to ice-cold DPBS with the antibiotics penicillin-streptomycin (400 U/mL and 400 µg/mL, respectively) and nystatin (240 U/mL). Incubation in this antibiotic-supplemented DPBS continued during the transport of the tissue to the Institute of Veterinary Physiology, Freie Universität Berlin (approximately 1 h).

### Explant culture

As previously reported [3,20], the subcutaneous adipose tissues were washed with fresh DPBS and cut into 2–3 mm^3^ blocks without visible blood vessels. The blocks were placed at equal distances of ~5 mm in 6-well cell culture plates with 0.5 mL/well DMEM with 4 mM L-glutamine, 20% FBS, penicillin-streptomycin (100 U/mL and 100 µg/mL), and amphotericin B (2.5 µg/mL), referred to as the base medium hereafter. After incubation in a humidified atmosphere at 5% CO_2_ and 37°C, a quantity of 1.5 mL pre-warmed base medium was added per well after the explants had attached to the bottom of each well (~48 h). The medium was replaced every 48 h, taking care that explant tissues were not washed away or floating on the medium. On the expansion of fibroblast-like cells, the explants were removed on the 7^th^ day, and cells were left to grow and become confluent. This cell culture is referred to as passage number zero (P0).

### Cell passaging

For the separation of cells from confluent ASC monolayers, the cells were washed with DPBS (without Ca and Mg) and incubated with 0.5 mL trypsin-EDTA (0.25% and 0.02%, w/v respectively) in PBS (without Ca and Mg) in a 5% CO_2_ incubator at 37°C for 10 min. After the detachment of the cells, 5 mL fresh DMEM medium with 10% FBS was added to stop the enzymatic trypsin action. The cells were transferred into T25 culture flasks supplemented with pre-adipocyte cell culture medium (DMEM with 10% FBS, penicillin- streptomycin (100 U/mL and 100 µg/mL), amphotericin B (2.5 µg/mL), and 15 mM HEPES). Cells were maintained in a humidiﬁed atmosphere of 95% air and 5% CO_2_ at 37°C. When cells were 85 – 90% confluent, ASC were trypsinized again, and this passage cycle was repeated in new T75 flasks until P5. Subsequently, the cells were counted manually in a Neubauer chamber and viability was assessed by the trypan blue exclusion test (> 95%). For the experiments, ASC (1.5 × 10^4^ cells/well) were transferred into 24-well cell culture plates with or without coverslips, the former being dedicated for immunohistochemistry staining, the latter for the measurement of lipid content and quantitative reverse transcription PCR. All further experiments were carried out with ASC at P6.

### Validation of ASC identity and multi-lineage potential

The ASC were kept in 24-well cell culture plates with coverslips and pre-adipocyte medium as described above until the cells had reached confluence with two additional days under conditions of growth arrest. Afterwards, the cells were immersed in adipocyte induction medium for 2 days. Adipocyte induction medium consisted of (if not stated otherwise) DMEM with 4 mM L-glutamine, supplemented with HEPES (15 mM), biotin (10 µM), bovine insulin (3 µg/mL), dexamethasone (0.3 µM), IBMX (0.1 mM), rosiglitazone (10 µM), penicillin-streptomycin (100 U/mL and 100 µg/mL, respectively), and amphotericin B (2.5 µg/mL). Thereafter, the medium was changed to differentiation medium, which consisted of DMEM with 4 mM L-glutamine, penicillin-streptomycin (100 U/mL and 100 µg/mL, respectively), amphotericin B (2.5 µg/mL), HEPES (15 mM), biotin (10 µM), and bovine insulin (3 µg/mL). Differentiation medium was applied for 7 or 14 days with culture medium exchange after every 48 h. The cells were washed with DPBS, fixed in 4% Roti-Histofix for 30 min, and assessed by phase contrast microscopy in order to evaluate the accumulation of triglycerides and phenotypic changes in cell morphology. The ASC were further examined using fluorescent immunocytochemistry staining against NT5E, THY1, ENG, DLK1, PDGFRα, GLUT4, FAS and FABP4 markers. The staining procedure and the use of specific antibodies are detailed in the next subsections.

To verify the multi-lineage potential of bovine ASCs, 1.35 × 10^5^ cells/well were plated in 6-well culture plates and treated with complete α-MEM. After reaching confluency, the medium was switched to osteogenic differentiation medium (complete α-MEM supplemented with 10 mM ß-glycerophosphate, 0.1 mM dexamethasone and 5 nM of 1,25-dihydroxyvitamin D_3_) for 21 days. Ascorbic acid (300 nM) was also present in the medium. The control cells were kept in complete α-MEM without osteogenic stimulants. After 21 days, culture medium was removed, cells were washed twice with DPBS, fixed with 4% Roti-Histofix for 30 min. The working solution of alizarin red stain (2 g alizarin red in 100 mL distilled water, pH 4.3) was added for 45 min at room temperature and cells were washed with DPBS. After staining, alizarin red was visualized using an inverted light microscope (Motic AE2000).

### Staining and evaluation of non-polar lipids

The lipid contents of the cells were assessed by Nile red staining. Briefly, the medium was removed from the culture wells, and the cells were washed twice with cold DPBS for 5 min. Thereafter, the cells were fixed in Roti-Histofix 4% solution for 30 min at room temperature and washed twice in DPBS for 5 min each. A final concentration of Nile red of 5 µg/mL (in DPBS) was used, in the dark for 5 min, to stain cellular lipids. Subsequently, the cells were treated with 0.5% Triton™ X-100 solution in the dark for 5 min and thereafter stained with 0.2 µg/mL of DAPI in the dark for 5 min. The fluorescence indices for Nile red and DAPI were measured by an EnSpire Multimode Plate Reader (PerkinElmer, Massachusetts, USA). For Nile red, two sets of wavelengths were used as suggested by Greenspan et al. [21]. These were (excitation/emission) 515/590 nm to visualize total lipids and 475/570 nm to selectively visualize non-polar lipids. For DAPI the excitation/emission was 358/461 nm. The fluorescence signal for Nile red (lipid index) was divided by DAPI fluorescence (nuclei index) to obtain a lipid/nuclei index in which the Nile red fluorescence was corrected for variation in cell density. The same staining protocols were applied to cells grown on 24-well plates for microscopic visualization of Nile red and DAPI fluorescence by using a Leica DMI 6000B epi-fluorescent microscope with a 20× objective (Leica microsystems, Wetzlar, Germany).

### Immunofluorescence cytochemistry

ASC were grown on round sterilized glass cover slips (6 mm). Using dual-colour immunocytochemistry, the cells were characterized for the presence of the ASC cell surface markers (NT5E, THY1, ENG, DLK1, PDGFRα) and markers of differentiated adipocytes (FABP4, GLUT4 and FAS). Briefly, the cells were washed with cold DPBS (with Ca and Mg), fixed in Roti-Histofix 4% solution for 30 min, and washed twice in DPBS for 5 min. Next, the cell membranes were permeabilized by incubation of cells with 0.3% Triton^TM^ X-100 in DPBS for 20 min, followed by an incubation in 5% goat serum for 25–30 min. Thereafter, the cells were incubated with primary and secondary antibodies as detailed in Table 1. Nuclei were counterstained with DAPI (0.2 µg/mL) at room temperature for 5 min in the dark. The cells were washed twice with DPBS, mounted on clean glass slides with Mount Fluor (BioCyc, Potsdam, Germany), and stored at 4°C. Negative controls were incubated with DPBS instead of the primary antibodies while the rest of the protocol remained unchanged. Fluorescence signals were evaluated on a Leica DMI 6000B epi-fluorescent microscope with a 63× objective.

**Table 1.** Characteristics and details of antibodies used, together with their antigens

Antigens	Primary antibodies	Incubation protocol (primary antibody)	Secondary antibodies*^a^*	Visualization (excitation/emission)
NT5E	Rabbit polyclonal, (ab137595), 1:100	At room temperature for 1 h and then left overnight at 4°C in a humidified chamber	Goat anti rabbit IgG, labeled with Alexa Fluor 488	489 nm/508 nm
THY1	Rabbit monoclonal, (ab92574), 1:50			
DLK1	Rabbit polyclonal, (ab21682), 1:200			
FABP4	Rabbit polyclonal, (ab85875), 1:100			
ENG	Mouse monoclonal, (MA5-11854), 1:50	At room temperature for 1 h and then left overnight at 4°C in a humidified chamber	Goat anti mouse IgG, labeled with Alexa Fluor 594	596 nm/620 nm
PDGFR-α	Rabbit polyclonal (orb6660), 1:50	At 37°C temperature for 1 h and then left overnight at 4°C in a humidified chamber	Goat anti rabbit IgG, labeled with Alexa Fluor 488	489 nm/508 nm
GLUT4	Rabbit polyclonal (ab33780), 1:30			
FAS	Rabbit polyclonal (ab22759), 1:50			

***^a^***Incubation with secondary antibodies was at room temperature for 45 min.

### RNA isolation and quantitative real-time polymerase chain reaction

For RNA isolation, the cultured ASC before induction and after the development of adipocytes (14 days) were washed two times with DPBS and trypsinized. Cells were resuspended in DPBS, centrifuged at 300 × *g* at 4°C for 5 min and the cell pellet was stored at −80°C in RNAlater® (Invitrogen, California, USA). The NucleoSpin® RNA kit (Machery-Nagel GmbH & Co., Düren, Germany) was used to extract total RNA according to the manufacturer’s instructions. The quantity of RNA was assessed at 260 nm by using a Nano-Photometer (Implen, Munich, Germany).

Total RNA (100 ng/µL) was reverse transcribed by using an iScript cDNA Synthesis Kit (Bio-Rad, Munich, Germany). Quantitative reverse transcription PCR was carried out in an Viia7 real time PCR cycler (Thermo Scientific, Massachusetts, USA) with SYBR Green master mix (Bio-Rad, Munich, Germany) and the gene-specific, intron spanning primers for *NT5E, THY1, ENG, PDGFRα, PPAR*γ, *LPL, GLUT4* and *FABP4* presented in Table 2. Amplification of cDNA was carried out in a final volume of 10 µL containing 5 µL mastermix, 1 µL primer sense, 1 µL primer antisense, and 3 µL cDNA. The temperature protocol consisted of an initial denaturation at 94°C for 3 min followed by 40 cycles of 94°C for 30 s, 58°C for 1 min, and 72°C for 30 sec. PCR was followed by a melting curve analysis to validate specificity. The C_t_ values of the target genes were normalized to ribosomal protein S19 (*RPS19*) and tyrosine 3-monooxygenase/tryptophan 5-monooxygenase activation protein zeta (*YWHAZ*). All reactions were performed in triplicate and relative gene expression was calculated by using the 2^−ΔΔCT^ method [22]. Negative controls without cDNA template were included in all reactions.

**Table 2.** Primers used to amplify specific genes of the bovine subcutaneous ASC and differentiated adipocytes

Gene	Sense (5ʹ-3ʹ)	Anti-sense (3ʹ-5ʹ)	Amplicon size (bp)	RefSeq identifier
*NT5E*	TTTGGAGGCACCTTTGACC	AGAGGCTCATAACTGGGCAC	212	NM_174129.3
*THY1*	CAACTTCACCACCAAGGATG	TCTGGATCAGCAGGCTTATG	140	NM_001034765.1
*ENG*	CCTCAGCGTGAACAAATCC	CGTGAAAGACCAGTTTGGAG	89	NM_001076397.1
*FABP4*	TGGGATGGAAAATCAACCAC	TGGCTTATGCTCTCTCATAAAC	112	NM_174314.2
*RPS19*	GGAAAAGGACCAAGATGGGG	CGAACGAGGCAATTTATTAACC	136	NM_001037467.2
*YWHAZ*	AGAGAGAAAATAGAGACCGAGC	AGCCAAGTAGCGGTAGTAG	144	NM_174814.2
*GLUT4*	AGCCATGAGCTATGTCTCC	AAGATGAAGAAGCCAAGCAG	255	NM_174604.1
*PPARγ*	TAAAGAGCCTGCGAAAGCC	GCTTCACGTTCAGCAAACC	156	NM_181024.2
*LPL*	AGAGTAAAGGCAGGAGAGAC	CAGCCAGACTTTCTATTCAGG	134	NM_001075120.1
*PDGFRα*	CATCTTTGACAACCTGTACACC	TAGAGTCCACCATCATGCC	113	NM_001192345.3
*GAPDH*	AAGAAGGTGGTGAAGCAGG	GCATCGAAGGTAGAAGAGTGAG	116	NM_001034034.2

### Experimental setups to optimize adipogenic differentiation

In total, three experimental setups were conducted to identify the best protocol for the rapid conversion of bovine ASC to mature adipocytes.

#### Experiment 1: optimization of the induction medium for bovine ASC

The first trial was carried out to investigate the minimal working concentration of adipogenic supplements in the induction medium. The basal induction medium was DMEM (with 4 mM L-glutamine) supplemented with FBS (10%), pantothenic acid (17 µM), biotin (33 µM), insulin (10 µg/mL), dexamethasone (1 µM), rosiglitazone (20 µM) and IBMX (250 µM) as stated by Riedel et al. [23]. This medium was referred to as the 100% medium. In parallel incubations, the concentrations of pantothenic acid, biotin, bovine insulin, dexamethasone, IBMX and rosiglitazone were reduced to 30% and 10% of the 100% medium, and the transformation of bovine pre-adipocytes into adipocytes was assessed by using Nile red staining of non-polar lipids.

#### Experiment 2: in vitro induction and differentiation potential of bovine ASC

To determine the adipogenic effects of FBS, BSL and AsA on ASC, a second experiment was conducted on subcutaneous ASC isolated from three different animals. In 24-well cell culture plates, confluent ASC that had undergone growth arrest were treated with 30% induction medium, i.e. DMEM (with 4 mM L-glutamine) supplemented with FBS (10%), HEPES (15 mM), biotin (10 µM), bovine insulin (3 µg/L), dexamethasone (0.3 µM), IBMX (0.1 mM), rosiglitazone (10 µM), penicillin-streptomycin (100 U/mL and 100 µg/mL, respectively), and amphotericin B (2.5 µg/mL). After 2 days of ASC induction, the cells were kept in seven different types of adipocyte differentiation media in which FBS, BSL or AsA were either present or absent for either 7 or 14 days. The code used to identify the treatments (TRT) is presented in Table 3. Adipogenic differentiation was assessed by using Nile red staining, immunocytochemistry and quantitative reverse transcription PCR.

**Table 3.** Experimental concentrations of ascorbic acid (AsA), foetal bovine serum (FBS) and bovine serum lipids (BSL) in the differentiation media used in Experiment 2

Treatments	AsA (µg/mL)	FBS (%)	BSL (µL/mL)
TRT-Con	0	0	0
TRT-1	0	10	0
TRT-2	0	10	10
TRT-3	0	0	10
TRT-4	40	10	0
TRT-5	40	10	10
TRT-6	40	0	10

#### Experiment 3: effect of AcA, glucose and various coatings on adipogenesis

ASC were cultured in 24-well cell culture plates that were either left non-coated or coated with collagen-A, gelatin-A or poly-L-lysine. After reaching confluence with two additional days under growth arrest, the ASC were kept in induction medium for 2 d. Two experimental runs with 4 replicates were conducted with various differentiation media based on the following components: DMEM without glucose but with L-glutamine (4 mM), penicillin-streptomycin (100 U/mL and 100 µg/mL, respectively), amphotericin B (2.5 µg/mL), HEPES (15 mM), biotin (10 µM), insulin (3 µg/mL), AsA (113 µM), and BSL (5 µL/mL). The treatment media were then supplemented with 10 or 25 mM glucose together with 0, 10 or 20 mM AcA. Adipogenic effects were determined by using Nile red staining after 7 and 14 d in differentiation medium.

### Statistics

All data sets presented in the manuscript were statistically analysed and graphs were plotted by using SigmaPlot 11.0 software (Systat Software Inc., San Jose, CA, USA). All experiments were conducted in triplicate with three to five different animals. Each observation was derived from duplicate (Experiments 2, 3) or triplicate (Experiment 1) measurements that were arithmetically pooled from 2 or 3 wells of a 24-well culture plate. Each animal was considered as an experimental unit. In Experiments 1 and 2, the effects of different supplements were investigated by two-way ANOVA with fixed effects of treatment and days of treatment, except for the quantitative reverse transcription PCR data, which was evaluated by one-way ANOVA. In Experiment 3, a three-way ANOVA was applied, and least square means were computed for the fixed effects of supplement treatments, coating materials, and days of treatment with their two- and three-way interactions. If ANOVA indicated a significant effect, differences among groups were identified by the Holm-Sidak or Dunn’s post-hoc test. All the data are presented as means ± standard errors of the mean (SEM); *P* < 0.05 was considered statistically significant.

## Results

### Verification of ASC identity

**Figure 1.**
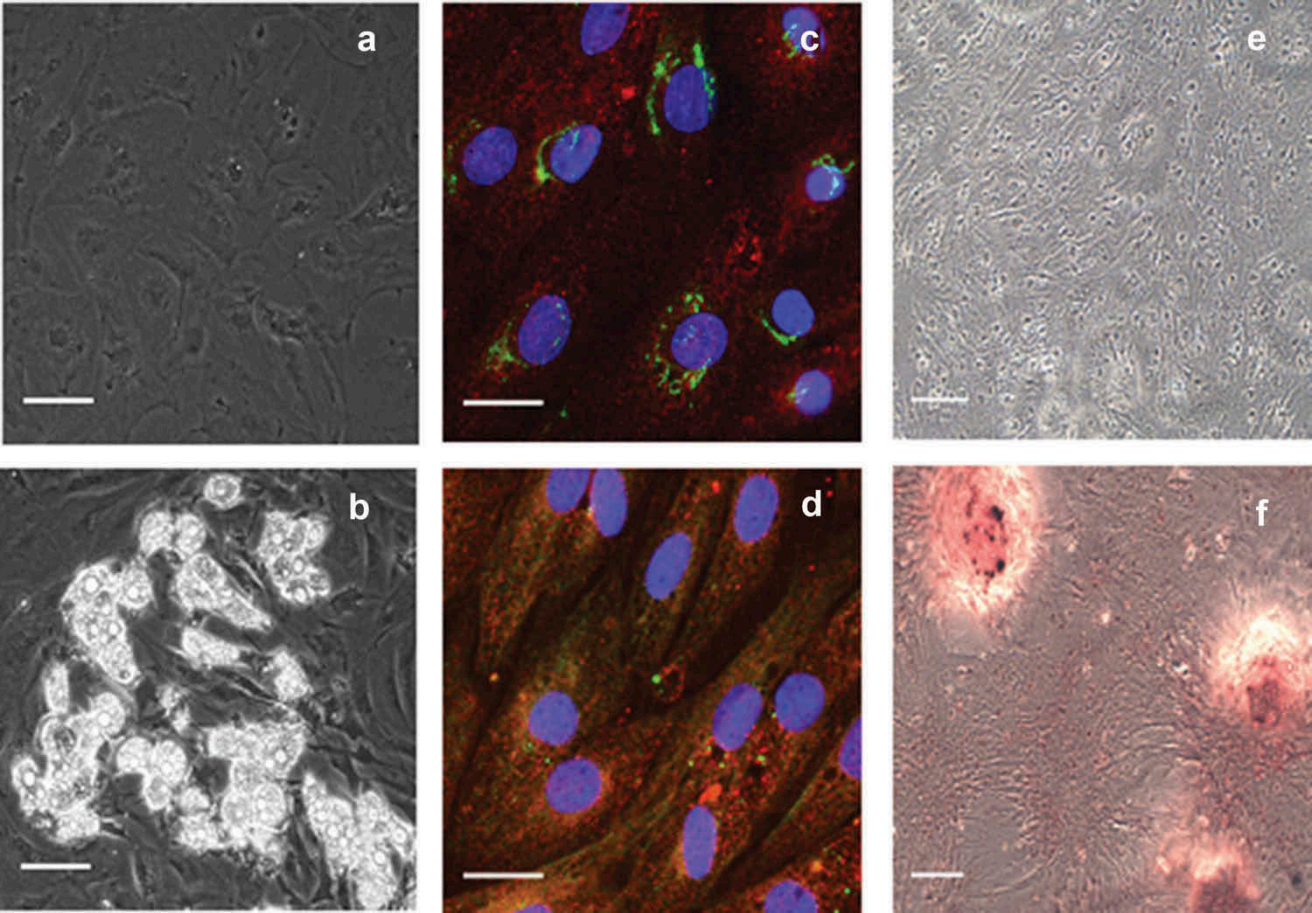
Verification of ASC identity. Representative phase contrast microscopic images of (a) pre-adipocytes before induction and (b) developed adipocytes after induction and 14 days in differentiation medium. Lipid droplets are amply present in differentiated adipocytes of graph b. The immunocytochemistry of undifferentiated pre-adipocytes identifies (c) the presence of NT5E (green) and ENG (red), as well as (d) recognition of THY1 (green) and ENG (red). For comparison, inverted light microscopic images using alizarin red staining are shown after 21 days (c) in control medium or (d) in osteogenic differentiation medium. The scale bar is representative of 100 µm in panels a and b (using a 20× objective), 25 µm in panels c and d (using a 63× objective), and 100 µm in panels e and f (using a 10× objective)

All adipose tissue explants were kept in 6-well tissue culture plates with a limited volume of culture medium to maintain them in permanent contact with the culture surface. Within 3–5 d, fibroblast-like cells started emerging from the tissue explants (Figure 1(a)). The cells were identified by their characteristic spindle shape. Upon transfer of the cells to induction medium, the ASC rapidly started to differentiate and to accumulate fat as shown in Figure 1(b).

For further identification of ASC, immunocytochemistry for well-defined ASC markers was carried out and revealed the presence of NT5E, THY1, and ENG (Figure 1(c,d)). NT5E was located around the nucleus and was most probably located in the Golgi apparatus, whereas the presence of THY1 and ENG was diffuse throughout the cell and in the cell membrane.

In addition, to demonstrate the multi-lineage potential of ASC, we differentiated the ASC to osteoblasts. Upon cultivation of the ASC in the appropriate differentiation medium, calcification was visualized using staining with alizarin red, a commonly used dye to stain calcium deposits. As shown in Figure 1, control cells incubated in the absence of osteogenic stimulants did not show any staining (Figure 1(e)) while cells kept in osteogenic differentiation medium accumulated minerals as demonstrated by dense staining that appeared red under the inverted light microscope (Figure 1(f)).

### Experiment 1: optimization of induction medium for bovine ASC

**Figure 2.**
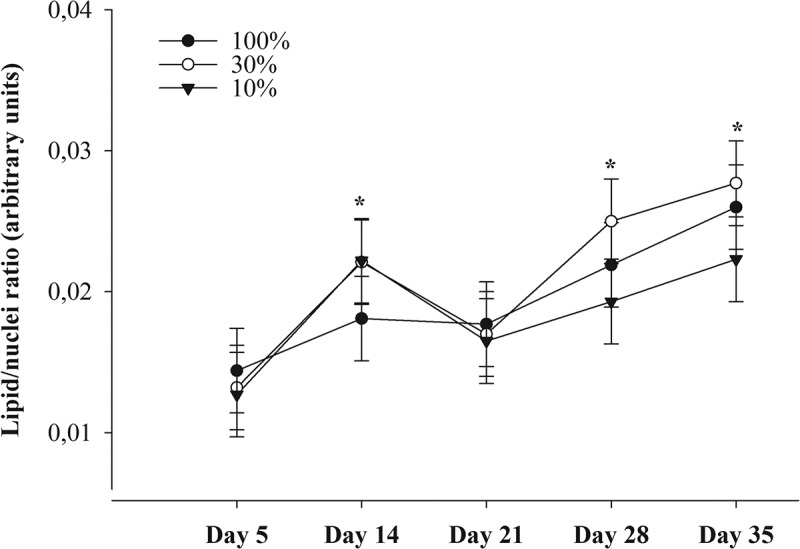
Influence of a gradual decrease in the concentrations of insulin, dexamethasone, rosiglitazone, 3-isobutyl-1-methylxanthine (IBMX) and biotin in the induction medium to 30% and 10% of their original concentrations on lipid incorporation by bovine ASC. The ‘100% medium’ contained 10 µg/mL insulin, 1 µM dexamethasone, 20 µM rosiglitazone, 250 µM IBMX and 33 µM biotin in the recipes used by Riedel et al. [22]. The results are presented as means ± SEM of three independent experiments with two replicates. *Asterisks indicate an effect of factor day with *P* < 0.05 to day 5

The induction of pre-adipocytes differentiation critically depends upon the parallel stimulation of insulin, glucocorticoid and peroxisome proliferator-activated receptor (PPAR)γ, and is greatly enhanced by cyclic adenosine monophosphate (cAMP) [24]. Thus insulin, dexamethasone, rosiglitazone, and cAMP-stimulating agents such as IBMX, are usually supplied in massively supraphysiological concentrations in induction media for adipogenic differentiation. To elucidate whether these extremely high concentrations are optimal for bovine ASC, we proportionally reduced the concentrations of supplements starting from ‘100% medium’ containing 10 µg/mL insulin, 1 µM dexamethasone, 20 µM rosiglitazone, 250 µM IBMX and 33 µM biotin to medium containing only 30% or 10% of each of these supplements. At all three concentrations, a rapid accumulation of cellular lipid occurred by day 5 (*P* < 0.001 to zero; Figure 2), indicating that only a few days in induction medium were sufficient for adipogenic induction in bovine ASC. Lipid accumulation between day 5 and day 35 was affected by the factor day of treatment (*P* < 0.001), with lipid accumulation on day 14, day 28, and day 35 being higher than that on day 5 (*P* < 0.05; Figure 2). The effect of treatment was not significant (*P* > 0.05); however, the ‘10% medium’ appeared to trigger lower lipid accumulation by day 28 and day 35 than the ‘30% medium’. Hence, we concluded that the reduction of induction supplements to 30% of those used by Riedel et al. [23] was the most effective way of achieving adipogenic induction in bovine ASC. We therefore used 3 µg/mL bovine insulin, 0.3 µM dexamethasone, 10 µM rosiglitazone, 100 µM IBMX and 10 µM biotin for induction in all the following experiments.

### Experiment 2: in vitro induction and differentiation potential of bovine ASC

**Figure 3.**
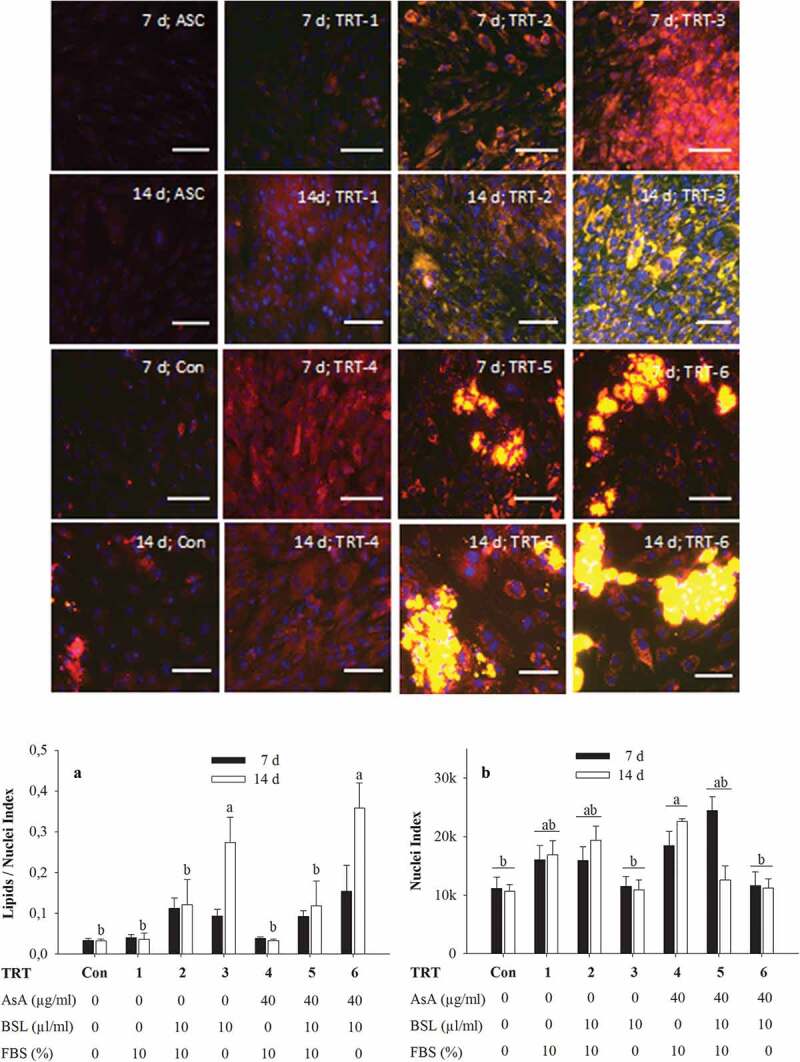
Nile red staining of lipids in adipocytes after 2 d in induction medium followed by 7 d or 14 d of incubation in various adipocyte differentiation media. Nile red was imaged at (excitation/emission) 515 nm/590 nm for total lipids (coded to red) and 475 nm/570 nm (emission) for non-polar lipids (coded to green). The green-red overlay results in bright yellow colour for lipid droplets. Nuclei were stained with DAPI and imaged at 358 nm/461 nm (emission). Scale bar = 100 µm (20× objective). The graph A shows quantification of non-polar lipids (475 nm/570 nm) relative to DAPI fluorescence (358 nm/461 nm) for three independent experiments with two replicates while graph B shows corresponding DAPI fluorescence. The data symbolize means ± SEM. ^a,b^Mean values with different superscripts are significantly different (*P* < 0.05). TRT, treatment; Con, negative control; AsA, ascorbic acid; BSL, bovine serum lipids; FBS, foetal bovine serum

To investigate the differentiation potential of bovine ASC, the cells were kept in induction medium developed in Experiment 1 for 2 d and further transferred into seven different types of differentiation medium, as described in Table 3 for either 7 or 14 d. The differentiation media affected lipid accumulation as shown by Nile red staining (Figure 3). Microscopically, the presence of non-polar lipids could primarily be seen following treatments TRT-2, TRT-3, TRT-5 and TRT-6, i.e. after treatments in which BSL was present; the presence of non-polar lipids was negligible in TRT-1, TRT-4 and the control treatment (TRT-Con). The lipids were visible as early as day 7 in the BSL-treated groups and appeared to become intensified at day 14, especially, in treatments in which FBS was absent (TRT-3 and TRT-6). Of note, the accumulation of non-polar lipids in the BSL-treated groups was greatly dependent on the presence of AsA. Despite a quantitatively similar content of lipids (see below), the pattern of lipid accumulation was mostly (albeit not exclusively) diffuse in the absence of AsA (TRT-2 and TRT-3). In contrast, the presence of AsA (TRT-5 and TRT-6) enhanced the build-up of larger fat vacuoles in defined clusters of adipocytes with other areas appearing devoid of lipids. The formation of the defined adipocyte clusters was linked to a change in the cellular shape of the fat-accumulating cells.

The quantitative statistical evaluation of non-polar lipid build-up in cells (in terms of lipids/nuclei ratio) is presented in Figure 3 graph a. The influence of the various treatments and of the factor day were both statistically significant (*P* < 0.001). Furthermore, a treatment × day interaction (*P* = 0.001) was detected that was based on a significantly enhanced lipids/nuclei ratio in TRT-3 and TRT-6 compared with all other treatments after 14 d of culture.

The incubation of cells in FBS-free medium not only enhanced the development of non-polar lipids in cells, but also inversely affected cell density (Figure 3 graph b). The DAPI fluorescence signal (representing the density of cell nuclei) was significantly affected by treatment (*P* < 0.001) and was lowest in treatments without FBS (TRT-Con, TRT-3 and TRT-6) but highest in the treatment regime with FBS and AsA (TRT-4), treatments TRT-1, TRT-2 and TRT-5 showing intermediate values. Neither day of treatment (*P* = 0.586) nor TRT × day interaction (*P* = 0.051) was significant.

**Figure 4.**
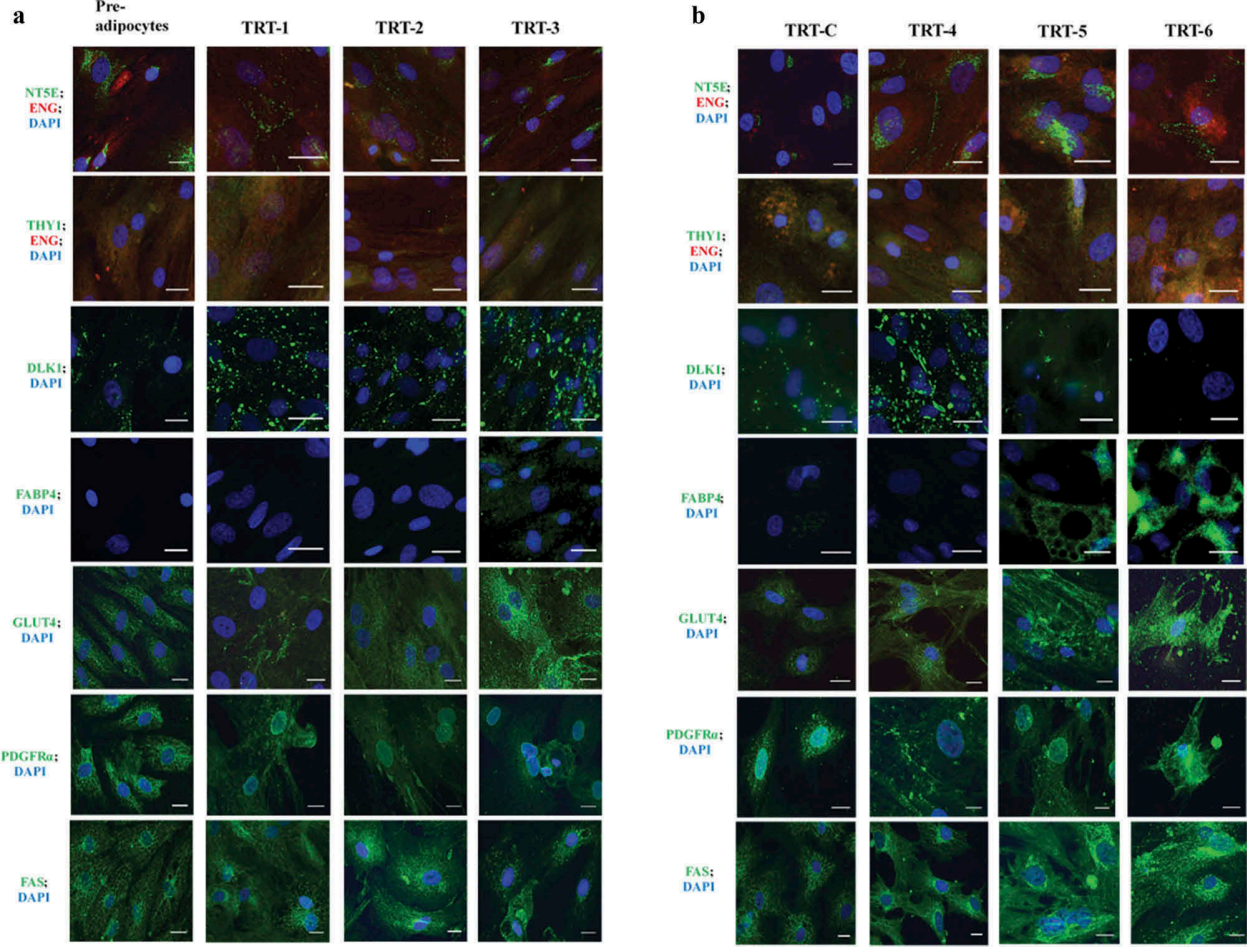
Immunocytochemical classification of bovine subcutaneous adipose-derived stem cells at different stages of development analysed for the presence of different ASC and adipocyte markers. All images were made after 14 d in different types of differentiation media (TRT-1 through TRT-6; for code see Table 3). The colour code of the antigen labels represents the fluorophore colour of the imaged antigen (red or green) and nuclear stain (DAPI, blue), respectively. Scale bar = 25 µm (63 × objective)

Immunocytochemistry was carried out in order to visualize the presence of cell-specific markers for ASC and adipocytes. Cells at the pre-adipocyte stage were positive for NT5E, THY1, ENG, PDGFRα, DLK1, GLUT4 and FAS (Figures 1 and 4), but FABP4 was absent (Figure 4). A visual increase in the development and intensity of the DLK1 surface marker occurred in the differentiation media (e.g. TRT-Con and TRT-1 to TRT-4). With an increase in culture age and the emergence of adipocytes, however, the DLK1 surface marker became less intense in the cells that were accumulating larger lipid droplets (TRT-5 and TRT-6). Cells with larger lipid droplets also showed a diverse round morphology plus evident FABP4 around the lipid droplets (Figure 4; TRT-5 and TRT-6). Cell surface markers NT5E, THY1, and ENG were present not only at the pre-adipocyte stage, but also during and after the differentiation process of ASC, but with a clear change in the intensity of these markers. The major change was observed in NT5E localization: at first, it was present around the nuclei, but with ageing and differentiation, it was found mainly on the cell surface (Figure 4). Immunostaining for PDGFRα was visible at all stages and found inside cells and on cell boundaries. With the progression of transformation towards adipocytes, the signal decreased. GLUT4 was present already at the preadipocyte stage and its intensity increased with the differentiation towards adipocytes. This increase was particularly evident in the treatments where BSL was present in the medium as shown in Figure 4. FAS was present throughout the experiments (Figure 4); however, a concentration of FAS signal within the Golgi apparatus was evident with the progression of cellular development towards adipocytes, again, especially in groups treated with BSL.

**Figure 5.**
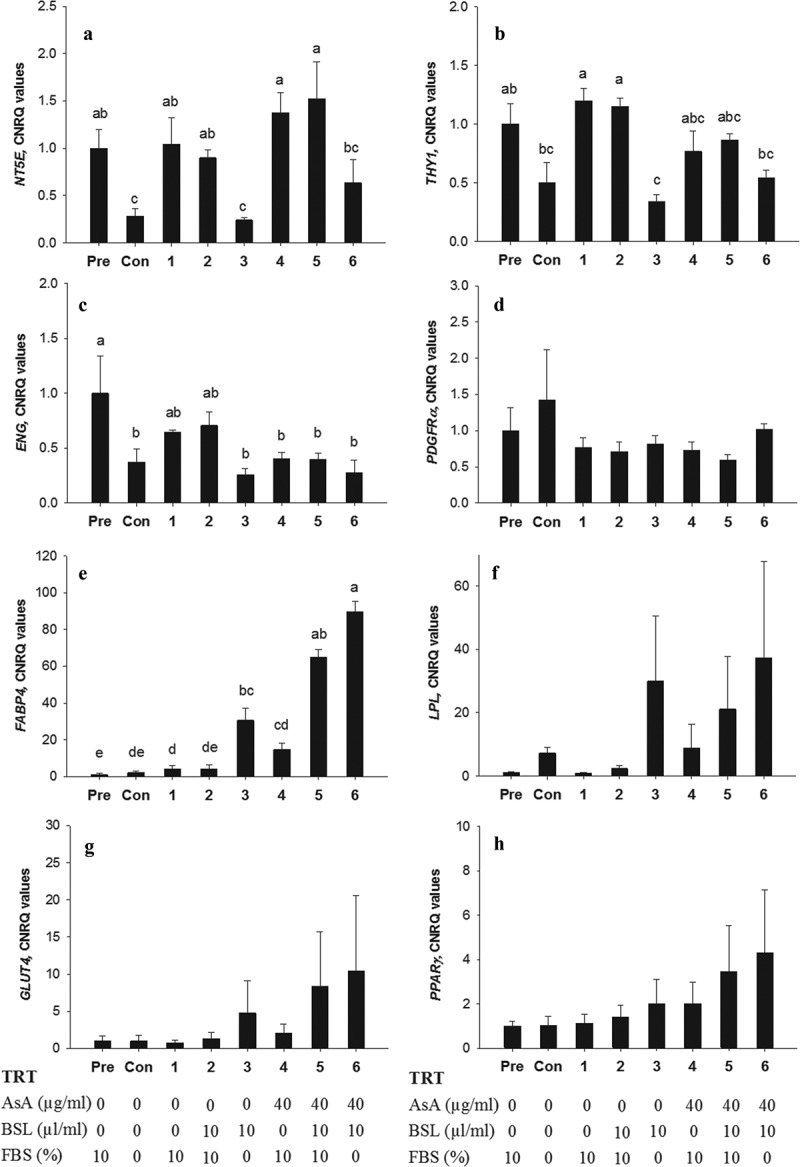
Relative mRNA expression for *NT5E, THY1, ENG, PDGFRα, FABP4, LPL, GLUT4 and PPARγ* in bovine subcutaneous ASC after *in vitro* transdifferentiation. Quantitative reverse transcription PCR analysis of *NT5E* (A), *THY1* (B), *ENG* (C), *PDGFRα* (D), *FABP4* (E), *LPL* (F), *GLUT4* (G) and *PPARγ* (H) are given as the mean ± SEM of four animals with three replicates. Pre, pre-adipocyte (RNA extracted before induction); Con, negative control; TRT = treatment; FBS, foetal bovine serum; AsA, ascorbic acid; BSL, bovine serum lipids. Letters (a-e) are used to denote statistical difference; columns within one graph are different if they do not share a common letter (*P* < 0.05)

To analyse the effects of the differentiation medium on the phenotypic changes in ASC further, cells were collected for reverse-transcription quantitative PCR analysis after 14 d in the seven different types of differentiation media. ANOVA identified significant changes in the mRNA abundance of the stem cell markers *NTE5, THY1, ENG* (*P* < 0.05). The Dunn’s test revealed that the expression of *NT5E* remained unaltered (*P* > 0.05) compared with pre-adipocyte values when FBS was supplemented in the differentiation medium but decreased significantly (*P* < 0.05) in FBS-free treatments (TRT-Con, TRT-3 and TRT-6; Figure 5(a)). The mRNA abundance for *THY1* was or tended to be lower (*P* < 0.05) in TRT-C, TRT-3, and TRT-6 in contrast with all other treatments (Figure 5(b)). The expression of *ENG* decreased or tended to decrease in all treatments compared with the pre-adipocyte stage with decreases being most pronounced in the FBS-free groups (TRT-Con, TRT-3 and TRT-6) and treatments with AsA (TRT-3 to TRT-5; *P* < 0.05; Figure 5(c)). Analysis of *PDGFRα* did not reveal any significant changes (Figure 5(d)).

The expression of the mature adipocyte marker *FABP4* increased when media were supplemented with BSL in the absence of FBS (TRT-3 and TRT-6, *P* < 0.05), however, the increase was two-fold higher in the additional presence of AsA (TRT-6, *P* < 0.05). In the presence of AsA, an increase in FABP4 expression was also seen when medium contained BSL and FBS (TRT-5; Figure 5(e)). The other mature adipocyte markers (*LPL, GLUT4*, and *PPARγ*) showed a behaviour similar to *FABP4*; however, due to high variance among animals, these changes were not significant (Figure 5(f–h)).

### Experiment 3: effect of AcA, glucose and various coating on adipogenesis

Based on the results of Experiment 2, we used a differentiation medium containing BSL and AsA but no FBS to test the hypothesis that the concentration of AcA in the medium has an effect on non-polar lipid accumulation, and that this effect is influenced by the availability of glucose in the culture medium. However, as the factor glucose was not significant, it was removed from the statistical analysis to enable three-way ANOVA with the factors AcA, day and coating, the last mentioned being used to test whether different coatings affect cell attachment/viability. None of the two- and three-way interactions among these factors were significant, and they are therefore not presented.

The factor coating affected the nuclei index (*P* < 0.001), which is indicative of cell density, with decreased values in non-coated and collagen-A-coated wells compared with poly-L-lysine or gelatin-A-coated materials (Table 4). Factor AcA also influenced the nuclei index (*P* < 0.001), with decreased values at 20 mM AcA compared with 0 and 10 mM AcA (Table 4). The nuclei index further changed with regard to factor day of treatment (*P* < 0.001), with slightly lower values after 14 d in differentiation medium as compared with 7 d (Table 4).

**Table 4.** Cell nuclei index and lipid/nuclei ratio of bovine ASC kept for 7 or 14 d intervals in plastic ware with various coatings and differentiation media containing various concentrations of acetic acid (AcA)

Nuclei index						
Factor*^a^*					SEM	P-value
Coating*^b^*	None	poly-L-lysine	Collagen-A	Gelatin-A		
	11724**^B^**	12846**^A^**	11767**^B^**	12612**^A^**	242	< 0.001
AcA	0 mM	10 mM	20 mM			
	12736**^A^**	12411**^A^**	11565**^B^**		192	< 0.001
Day	7 d	14 d				
	12892**^A^**	11583**^B^**			157	< 0.001
Lipid/Nuclei ratio						
Factor					SEM	P-value
Coating	None	poly-L-lysine	Collagen-A	Gelatin-A		
	0.035	0.029	0.035	0.033	0.004	0.741
AcA	0 mM	10 mM	20 mM			
	0.029	0.034	0.036		0.004	0.438
Day	7 d	14 d				
	0.026**^B^**	0.041**^A^**			0.003	< 0.001

Non-polar lipids were measured after Nile red staining (excitation/emission, 475 nm/570 nm), whereas nuclei were assessed by DAPI fluorescence (excitation/emission, 358 nm/461 nm).

*^a^*Factor glucose was removed from the analyses after verification that the different glucose concentrations had no significant effect.

^A,B^Values with similar capital letters in a single row do not differ statistically at *P* < 0.05.

The lipid/nuclei ratio, indicative of the accumulation of lipid droplets, was only affected by the factor day of treatment (*P* < 0.001), with higher values after 14 d in the differentiation medium as compared with 7 d (Table 4).

## Discussion

Adult adipose stem cells are an important source of MSC and can be conveniently recovered and efficiently used in regenerative therapies because of their immune-compatible properties. In many species, ASC have been isolated and studied for their multi-linage differentiation, e.g. in man [25], cattle [20] and mouse [26]. The removal of pre-adipocytes from adipose depots is an easy cost-effective process and does not require co-incubation with toxic chemicals for cellular differentiation. In preliminary experiments, we found that ASC are especially abundant in younger animals. Therefore, we used calves of < 11 months of age for the explant cultures, adipose tissue was isolated caudally to the mandible, in the region of the 2^nd^ to 3^rd^ vertebrae, immediately after conventional slaughter. The harvested ASC had a fibroblast-like shape when they were in the growth phase but became round to oval when transdifferentiating into functional adipocytes. The differentiation and shape of cells depends on many important factors such as the initial plating concentration of cells [27] and the commitment of cells towards adipogenesis through the increased expression of the *DLK1, FABP4* and *PPARγ* genes. The use of an induction media cocktail seems to be necessary, especially for the activation of *PPARγ* and its downstream genes. PPARγ is a member of the nuclear steroid receptor family and is activated when bound to natural long chain fatty acids. Previous studies carried out on primary ASC have shown an increase in the mRNA of *PPARγ* during the process of differentiation [28]. Although the increase in the mRNA of *PPARγ* was not significant in the present study, the treatments that accumulated lipids most intensely, at least, appeared to have a higher expression of *PPARγ*, especially treatments receiving BSL and AsA.

Substances that activate PPARγ (e.g. thiazolidinedione and rosiglitazone) act as potent stimulators of ASC differentiation [29]. The presence of dexamethasone in the induction medium stimulates the expression of *PPARγ* and other nuclear factors, and ASC can progress towards transdifferentiation [30]. However, the use of dexamethasone for extended periods of time and in excessive amounts can be detrimental for ASC induction and differentiation [31]. Moreover, insulin has a well-known adipogenic effect and is therefore often used to activate various metabolic genes that finally trigger adipogenesis, including *PPARγ* [31]. Hyperinsulinic concentrations in the culture medium are therefore helpful to induce transdifferentiation.

Published protocols for the induction of adipogenic transdifferentiation regularly use excessively high concentrations of PPARγ agonists, dexamethasone and insulin, together with IBMX and biotin. To elucidate whether such elevated concentrations are too high for bovine ASC and induce negative effects on adipogenic transdifferentiation, we first performed a titration experiment in which we reduced the concentration of such supplements to 30% and 10% of their initial values. Our results demonstrate no substantial difference in the induction of transdifferentiation among the three concentrations tested. Since the 30% medium performed numerically (although not statistically) best over a prolonged period of time, we used and recommend this intermediate concentration of supplements for further experiments.

We further aimed at characterizing the marker proteins of ASC in Experiment 2. We had previously reported the immunocytochemical characterization of bovine subcutaneous ASC [20]. In that previous study, we focused on the prominent effects that BSL elicit on adipocyte differentiation and on the changes of cellular markers during that process. In the present investigation, we additionally used the quantification of Nile red fluorescence to measure lipid accumulation. This approach verified that the addition of BSL without FBS in the differentiation medium significantly improved the accumulation of non-polar lipids in cells, suggesting that serum-free medium and BSL enhanced the adipogenic potential of ASC. Bovine serum lipids are known to augment the internalization of long chain fatty acids in the presence of insulin via FABP4 [32]. The internalized fatty acids can then increase adipogenesis by acting as natural ligands for PPARγ and by serving as a direct fatty acid source for triglyceride synthesis.

Among the important mesenchymal stem cell markers that change during adipogenesis, the expression of ENG depends on the stem cell source (bone marrow or adipose tissue) and the period of maintenance in the culture medium [33]. As an extension of our previously reported results, the present study revealed a higher amount of ENG at pre-adipocyte stage, evidenced both by immunocytochemistry and by quantitative reverse transcription PCR. In addition to ENG, the cultured pre-adipocytes stained positively for NT5E and THY1. These results are in agreement with the results of Jones et al. who further demonstrated that the presence of ENG, NT5E and THY1 occurs irrespective of their passage number in culture [34]. In human mesenchymal stem cells (MSC), the positive expression of ENG, NT5E and THY1 is taken as evidence for the pluripotency of stem cells [35]; however, Boxall and Jones [36] have noted the absence of THY1 in MSC of goats and sheep. Our results showed the noticeable expression of NT5E, THY1, and ENG protein even at later stages of bovine subcutaneous ASC differentiation to adipocytes. This similarly applied to *PDGFRα* where immunocytochemical experiments showed an only mild decrease in signal strength during differentiation with no changes in mRNA abundance. Therefore, the use of these markers to identify cells as being potent for lineage differentiation is debatable. In agreement with this suggestion, Mark et al. [37] have reported that only 15% of human bone marrow cells have the potential to differentiate towards the adipogenic lineage rather than osteogenic lineage, as based on the presence or absence of ENG.

The omission of FBS from the incubation medium in the present study decreased the nuclei index, i.e. stopped cell proliferation, and was associated with the lower mRNA expression of *NT5E* and *ENG*. However, cells undergoing the FBS-free treatments (TRT-Con, TRT-3 and TRT-6) still had clearly detectable mRNA and protein levels for NT5E and ENG. Therefore, the expression of the two markers should not be linearly associated with MSC renewal or MSC renewal capability. The changes in the mRNA expression of *THY1* largely mirrored those of *NT5E* and *ENG*. This supports recent findings that *THY1* expression decreases with the differentiation of 3T3-L1 cells into functional adipocytes [38], and that knockout of *THY1* predisposes mice to adiposity [38,39].

In contrast to the ASC marker genes, the mRNA expression of *FABP4* was induced by the absence of FBS and in the presence of BSL in the differentiation medium. A striking finding was that the supplementation with AsA greatly enhanced the expression of *FABP4*. The latter was even true for media containing FBS (TRT-5) or being devoid of BSL (TRT-4). The combination of BSL and AsA (in the absence of FBS; i.e. TRT-6) was most effective in inducing the mRNA expression of *FABP4*. Cuaranta-Monroy et al. [40] have previously demonstrated the positive effect of AsA on the expression of *FABP4* in adipocytes differentiated from mouse embryonic stem cells. Our study extends these findings by demonstrating that the increased expression of *FABP4* is linked to the greater lipid accumulation in selected *FABP4*-containing cell clusters but not necessarily the greater lipid accumulation by the whole cell culture. Such clusters were typical for TRT-5 and TRT-6, which exhibited the highest expression of *FABP4* and visually contained many functionally mature adipocyte clusters.

The cellular function of FABP4 is the coupling of fatty acids to several molecular targets as a fatty acids chaperone [41]. Via peroxisome proliferator response elements, the transcription of *FABP4* is directly coupled to PPARγ and is thus prominently induced during ASC differentiation [42]. Vice versa, FABP4 appears to be a negative feed-back master regulator of PPARγ and plays a crucial role in the ubiquitination of PPARγ and, consequently, the down-regulation of insulin sensitivity in the mature adipocyte. The latter is seen as a major cause of morbidities related to insulin resistance [43]. Thus, the present study leads to the valuable conclusion that adipocyte differentiation and the expression of *FABP4* are regulated by AsA independently of the global lipid accumulation of an ASC culture.

Ono et al. [44] have reported that AsA stimulates the synthesis of procollagen and accelerates the differentiation of adipocytes, the latter being evidenced by the development of larger intracellular lipid droplets. Our results confirm that AsA has indeed a synergistic effect in the transformation of cells, although our results demonstrate that BSL is the most important supplement for the accumulation of non-polar lipids in bovine adipocytes, whereas AsA does not have a significant influence on the quantity of intracellular non-polar lipids, when BSL is present. To our knowledge, this is the first study reporting the dissimilar role of AsA and BSL for the amplification of *FABP4* with synergistic effects on adipogenic differentiation.

The antioxidant capacity of AsA might play a central role in this process. An earlier study by Lee et al. [45] has demonstrated that a plant-derived antioxidant (puerarin) also enhances differentiation and the upregulation of the expression of *FABP4* in 3T3-L1 cells. Furthermore, the knockout of *FABP4* decreases oxidative stress and uncouples obesity from inflammation in macrophages [46]. Together with the findings of the present study, these results support the conclusion that FABP4 plays a central role in the redox status of the cell; this is not entirely explainable by the degree of lipid accumulation. Vice versa, the redox status has a negative feedback on the expression of *FABP4* itself.

The treatment conditions leading to the largest amounts of FABP4 had the lowest abundance of DLK1 protein. This agrees with the known role of DLK1 as a negative regulator of adipocytes differentiation [47]. DLK1 is known to bind to fibronectin in the extracellular matrix; the fibronectin-DLK1 complex then interacts with integrin receptors to inhibit adipogenesis via the mitogen-activated protein kinase/extracellular-signal regulated kinases (MEK/ERK) pathway [48].

In addition to *FABP4*, we also analysed gene expression of other functionally relevant genes of mature adipocytes, namely, glucose transporter type 4 (*GLUT4*) and of lipoprotein lipase (*LPL*). *GLUT4* mediates the insulin-stimulated uptake of glucose into adipocytes and skeletal muscles [49]. Immunocytochemical staining confirmed the presence of GLUT4 in pre-adipocytes and all other treatment conditions with increasing intensity in treatment conditions that favoured the development of mature adipocytes (TRT-5 and TRT-6). Also quantitative real-time PCR indicated that highest values are associated with these two treatment conditions. However, in contrast to *FABP4*, we detected high variability in the expression levels between the individual animals. The same was true for the expression of *LPL* for which the expression pattern was again very similar to *FABP4*. Unfortunately, we could not identify a suitable antibody recognizing bovine LPL by immunocytochemistry. We therefore decided to investigate protein expression of fatty acid synthase (FAS), another enzyme with prominent role in triglyceride accumulation. FAS expression was identified under all treatment conditions with strongest staining in culture media that contained AsA as well as BSL (TRT-5 and TRT-6) that promoted the differentiation of ASC to mature adipocytes most effectively.

A special feature of bovine adipocyte metabolism *in vivo* is that AcA rather than glucose is the principal source of fatty acid synthesis [19]. Therefore, we tested the effects of various glucose and AcA concentrations on ASC differentiation in Experiment 3. With regard to the conventional glucose concentration in cell culture media, namely 25 mM, our results showed that a lowering of the glucose concentration to 10 mM did not have any significant effect on ASC lipid incorporation at various levels of AcA. Increasing the concentration of AcA to 10 and 20 mM induced no significant effects of AcA on lipid storage although lipid accumulation was numerically higher. All in all, this means that a glucose concentration of 10 mM does not limit the adipogenic transdifferentiation of bovine ASC and that AcA may have a stimulating effect on this process that requires further investigation. Nonetheless, an AcA concentration of 20 mM can lead to a decrease in nuclei density, i.e. cell number, and may thus be potentially too high and elicit detrimental effects on the cultured cells.

A valuable methodological result of Experiment 3 was that the coating of the culture dishes with poly-L-lysine and gelatin-A was associated with higher nuclei indices. This increase can be taken as an indication that cell survival and adherence improve if the cells are grown in the presence of these coatings. Experiment 3 also verified an increase in the nuclei ratio between 7 and 14 d, as similarly observed in BSL-containing, ASC-containing and FBS-free medium in Experiment 2, and thus identifies such a medium composition as highly suitable for the differentiation of bovine ASC-derived adipocytes.

In conclusion, this study shows that the differentiation and adipogenesis of bovine subcutaneous ASC-derived adipocytes is optimal in poly-L-lysine or gelatin-A coated culture wells, in the absence of FBS but in the presence of BSL and AsA. The last mentioned appears to have a unique role in *FABP4* induction, although this function is not linearly related to the primarily BSL-driven lipid accumulation and is more likely to be associated with the redox properties of AsA. The study further indicates that AcA may stimulate lipid accumulation in ACS-derived adipocyte cultures. However, the latter effect may be difficult to uncover in the presence of BSL and glucose as alternative fuels for adipogenesis and therefore requires further investigation.
